# Efficient B Cell Depletion via Diphtheria Toxin in CD19-Cre/iDTR Mice

**DOI:** 10.1371/journal.pone.0060643

**Published:** 2013-03-27

**Authors:** Filiz Demircik, Thorsten Buch, Ari Waisman

**Affiliations:** 1 Institute for Molecular Medicine, University Medical Center of the Johannes-Gutenberg, University of Mainz, Mainz, Germany; 2 Institute for Medical Microbiology, Immunology and Hygiene, Technische Universität München, Munich, Germany; French National Centre for Scientific Research, France

## Abstract

B cells were first discovered as antibody producing cells, as B-1 B cells and finally as effector cells. In recent years their capacity to serve as antigen presenting cells is increasingly appreciated, and better tools are needed to study their function. We have previously described a new mouse model, the iDTR mice, that allow for the Cre-mediated expression of the diphtheria toxin receptor, thus rendering cells that express the Cre-recombinase sensitivity to diphtheria toxin. Herein we describe a new mouse line, the B-DTR mice, where the CD19-Cre was crossed to the iDTR mice. B-DTR allows for the efficient and cost-effective depletion of different B cell subpopulations, but only partially plasma cells. These mice can therefore be used to study the importance of B cells versus plasma cells in different immune responses and autoimmune diseases.

## Introduction

The B cell lineage participates in immune responses through various means, including cytokine secretion, antigen presentation and production as well as secretion of antibodies. Depletion of B cells has proven useful in the treatment of autoimmune diseases. It results in the reduction of autoantibodies [1]–[5] but also affects autoimmune diseases through unknown mechanisms as seen in multiple sclerosis [6]–[9]. In addition, B cell depletion is used as therapy in lymphomas [10]–[15]. Consequently, nowadays depletion of B cells is a common therapy in clinical routine and especially anti-CD20 antibodies are commonly used [16]–[18]. Immunoglobulins are secreted by B-1 cells and professional antibody-secreting plasma cells, but plasma cells do not express classical B cell surface molecules including CD20 and therefore avoid depletion by CD20-specific monoclonal antibodies. A depletion of plasma cells would be advantageous to mediate a decrease of serum immunoglobulin.

Animal models are ideal to evaluate B cell depletion mechanisms and depletion efficiency. We have previously generated a mouse line in which the simian diphtheria toxin receptor (DTR) gene can be expressed after the cross to Cre-recombinase expressing mice [19]. We have shown that cross of these mice, termed iDTR, to mice that express Cre in B cells (CD19-Cre) results in mice with B cells expressing DTR, and thus are made sensitive to depletion following injection with diphtheria toxin (DT). Following Cre-mediated deletion of a transcriptionally stop cassette, DTR is expressed by the ubiquitous *gt(ROSA26)Sor* (R26) locus. Additionally, this system serves as a “genetic memory”, as after the recombination event the genome stays recombined and the DTR is transcribed also if B cells further differentiate to plasma cells [20]. The cross of the iDTR mice to the CD19-Cre can therefore serve as a model to deplete B cells in a cost-effective long term way.

We set to use these mice to effectively deplete B cells and plasma cells. Therefore we started with an evaluation of the efficiency of the recombination of CD19-Cre knockin mice [21], crossed to an eYFP reporter strain [22], in which the EYFP cassette is inserted at the same position as the DTR cassette in the iDTR mice. Using the iDTR/CD19-Cre system we found efficiency of up to 99% depletion of different B cell subpopulations, when the mice were treated by intra peritoneal injections of a daily dose of 25 ng DT per gr bodyweight for 4 days. This treatment was more effective to deplete mature B cells, immature B cells in BM exhibited the lowest depletion rate until this population leaves the BM.

## Results

**Figure 1:**
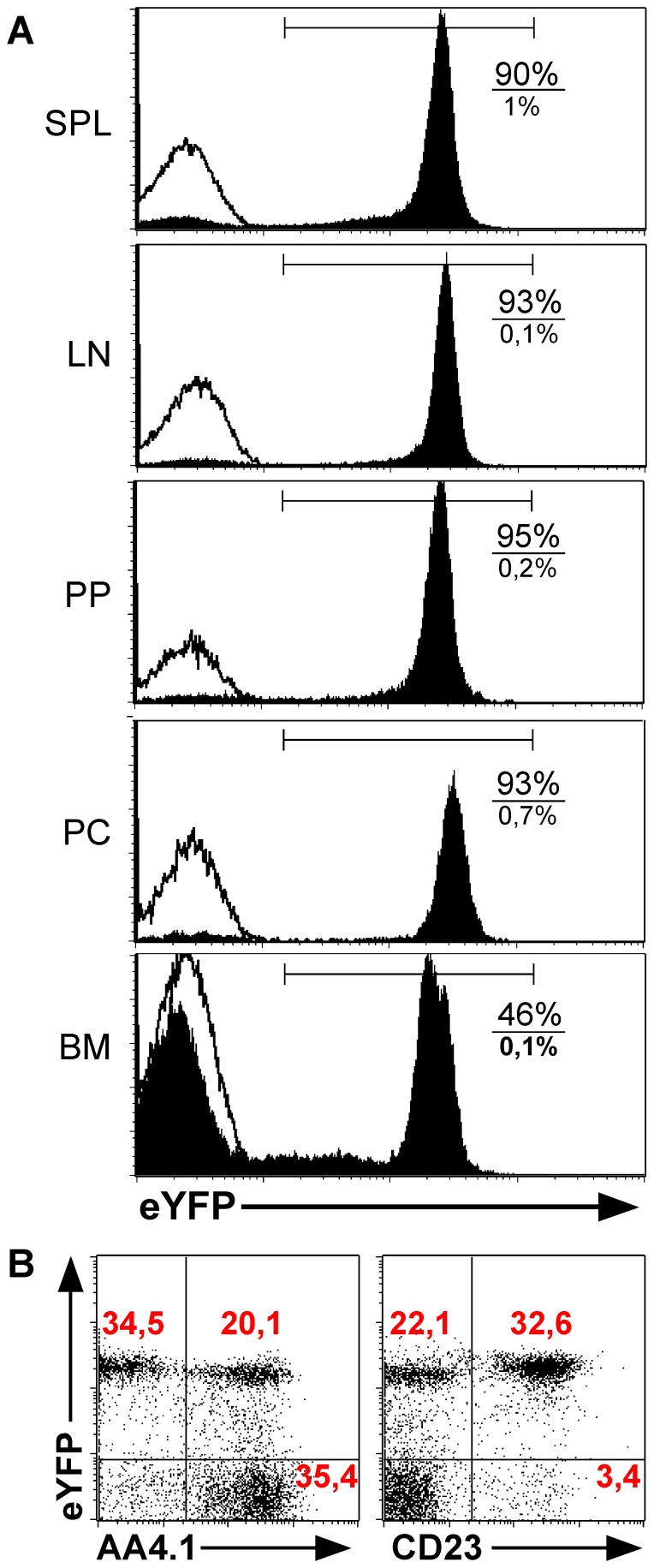
eYFP expression in CD19-Cre/eYFP mice. A. CD19-Cre/eYFP mice were analyzed for eYFP expression in the B cell compartment of spleen, lymph nodes, Peyer's patches, peritoneal cavity and bone marrow. The shown histograms are gated on live lymphocytes and CD19. The filled histogram curve represents CD19-Cre/eYFP mice and the unfilled control mice. Percentages of eYFP^+^ CD19^+^ cells are shown in the histograms, upper number/percentage B-DTR mice, lower panel shows cells of control mice. B. To identify the CD19^+^ eYFP^−^ B-cell fraction in bone marrow of CD19-Cre/eYFP mice, bone marrow cells were stained for AA4.1 and CD23.

The course of different autoimmune diseases, including rheumatoid arthritis, multiple sclerosis and others benefits from the depletion of B cells [23]–[28]. The reason for this beneficial effect of B cell elimination is not completely understood, and may result from a harmful effect of antibodies or other potential pathogenic roles of these cells, which need to be elucidated. To better understand how B cell depletion reduced autoimmune diseases, it is important to use an effective and low-cost system. A system to achieve this is our previously published iDTR system [19], in which the DTR can be conditionally expressed upon a cross to a Cre-recombinase expressing mouse line. For DTR expression by B cells, we used the CD19-Cre mice, which were shown to effectively recombine target genes in a B cell specific manner [21]. The verification of the CD19-Cre expression in all B cell subpopulations was done with the help of the eYFP reporter mouse strain. Both, the EYFP gene and the DTR coding region were inserted in the same locus under the control of the promoter of the R26 locus. We found EYFP expression in 90–95% of the B cells isolated from spleen, lymph nodes, Peyer's patches and peritoneal cavity. In contrast, only 46% of the CD19^+^ B cells in bone marrow express EYFP, as can be seen in Fig. 1A. This cell population was therefore investigated in more detail, and we found that the majority of the EYFP expressing cells in the BM are AA4.1^−^, thus mature B cells (Fig. 1B). These findings suggest that it takes time after the cells start to express CD19, consequently until they express sufficient amounts of the Cre-recombinase, which then removes the STOP cassette in the R26 locus resulting in EYFP expression. As all these processes take time, early B cells in the BM are EYFP negative and are therefore predestined to express little or no DTR.

**Figure 2:**
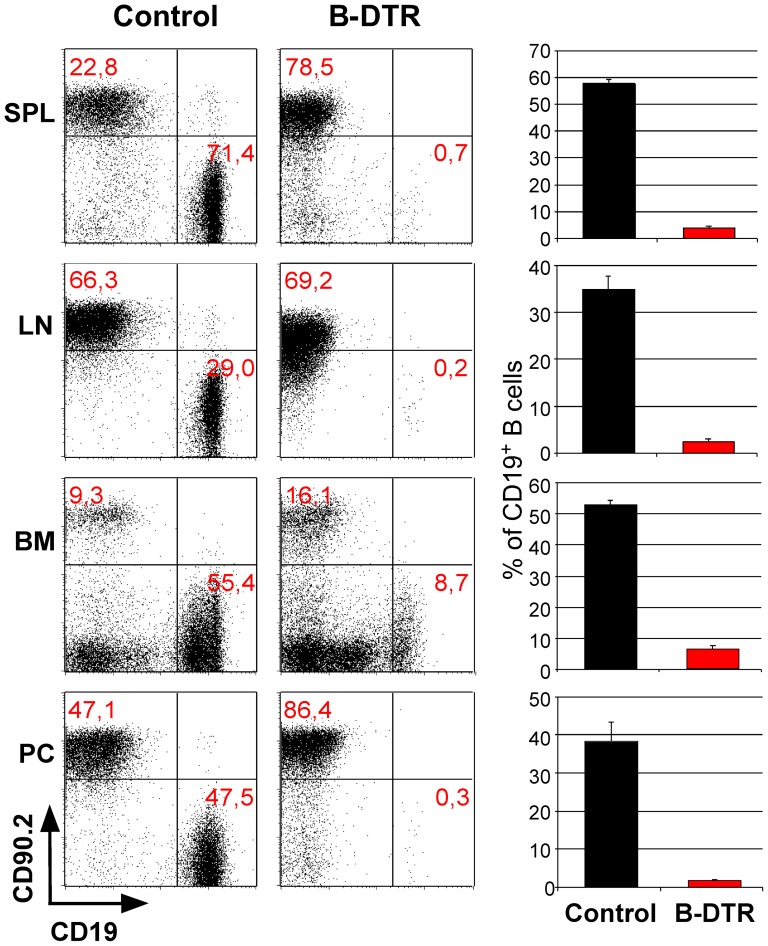
Diphtheria toxin mediated depletion of B cells in B-DTR mice. The mice were treated once daily for 4 days with a dose of 25 ng/g body weight of diphtheria toxin. The decrease of B cells in comparison to T cells is shown in spleen, lymph nodes, bone marrow and peritoneal cavity; dot blots in the left panel and the corresponding bar charts in the right panel.

Next, we crossed the CD19-Cre mice to the iDTR strain resulting in mice with B cells expressing the DTR (termed B-DTR). We utilized different protocols to optimize the B cell depletion via intra peritoneal diphtheria toxin (DT) application (not shown), finally choosing the most effective one, namely treatment with 25 ng per gram of body weight, for four consecutive days. One day after this treatment we could achieve almost complete depletion of B cells in the spleen, lymph nodes and peritoneal cavity. No change in B cell numbers was observed in control mice (single transgenic for the Cre or the iDTR alleles) after a similar treatment (Fig. 2). The only organ with a less effective depletion was the bone marrow, as can be seen in Figure 2. As already suggested above in Fig. 1 for the CD19-Cre/R26eYFP mice, it is highly plausible that time does not suffice for effective DTR expression in all early B cells in the BM.

**Figure 3:**
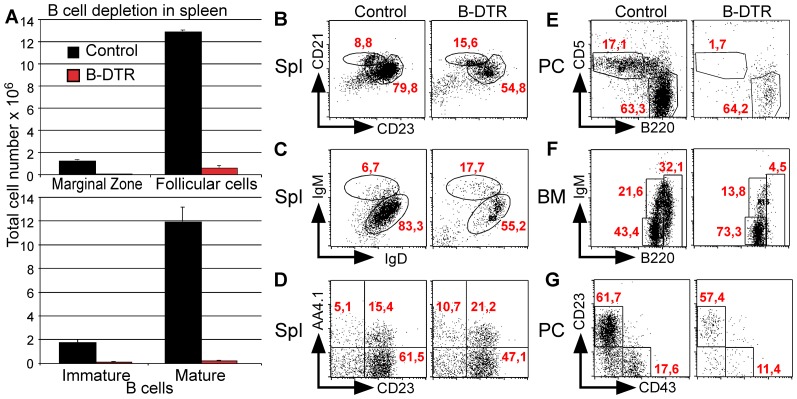
Efficiency of B cell depletion. A. Number of marginal zone, follicular, immature and mature B cells in spleen. B. Depletion of B cells in bone marrow, gated for B220^+^. C. Depletion of transitional B cells in spleen, gated for CD19^+^ cells. D. Depletion of mature and immature B cells in spleen, gated for CD19^+^ cells. E. Depletion of marginal zone and follicular B cells gated for CD19^+^ cells. F. Depletion of B1a and B2 B cells in peritoneal cavity, gated for CD19^+^. G. Depletion of resting B cells in peritoneal cavity gated for CD19^+^ the percentages of cells in the different shown B cell subpopulations are indicated.

To analyze the depletion efficiency in sub-populations of B cells, B-DTR and control mice were treated according to the above-described protocol. Again the depletion in spleen was almost complete, reflected in total cell numbers: hardly any CD19^+^ cells were left (Fig. 3A). Among the remaining cells, we found that the depletion of mature B cells was more effective compared to immature B cells. As can be seen in Fig. 3B in the few remaining B cells, the percentage of mature IgD^+^IgM^+^ B cells decreased from 83,3%±0,59% to 55,2%±2,3%, in contrast the percentage of the immature IgD^−^IgM^+^ B cells, which increased from 6,7% to 17,7%. Similarly, we could show that the ratio of mature to immature/transitional B cells, as seen by staining with AA4.1 shifted towards the immature AA4.1^+^ CD23^−^ T1 B cells in control mice 2,8%±0,4% and in B-DTR mice 13,1%±0,8%, indicating that these cells are less susceptible to depletion (Fig. 3C). Interestingly, also the ratio between follicular and marginal zone B cells changed after depletion: While marginal zone cells constituted a bit less than 10% (8,66%±0,31%) of splenic B cells in control mice, their proportion almost doubled (17,5%±0,83%) after depletion (Fig. 3D). The latter may indicate that marginal zone B cells might be less susceptible to DT-depletion compared to the conventional splenic B cells. As expected by the analysis of EYFP expression and the already shown depletion, the ablation of immature B cells in the BM was not complete, but among the mature B cells (B220^high^) the B cell depletion was almost complete, from 37%±1,6% in control mice only 1,2%±0,5% in B-DTR mice (Fig. 3E)

Following DT application depletion in peritoneal cavity (PC) was almost complete, and very few B cells were left. Further, the balance was tilted towards the B-2 cells. As can be seen when stained for CD5, the CD5^+^ population is decreasing from 17,7%±2,1% in control mice to 2,1%±0,8% in the depleted mice, while the percentage of B220^+^ B cells is nearly constant, control mice 46,7%±4,8% and depleted mice 50,3%±5,4% (Fig. 3F). Also staining for CD43 revealed that B-1 cells are more sensitive to depletion compared to B-2 cells (Fig. 3G). Thus, we conclude that our system allow excellent depletion of B cells also in the peritoneal cavity, particularly of the B-1 B cells.

**Figure 4:**
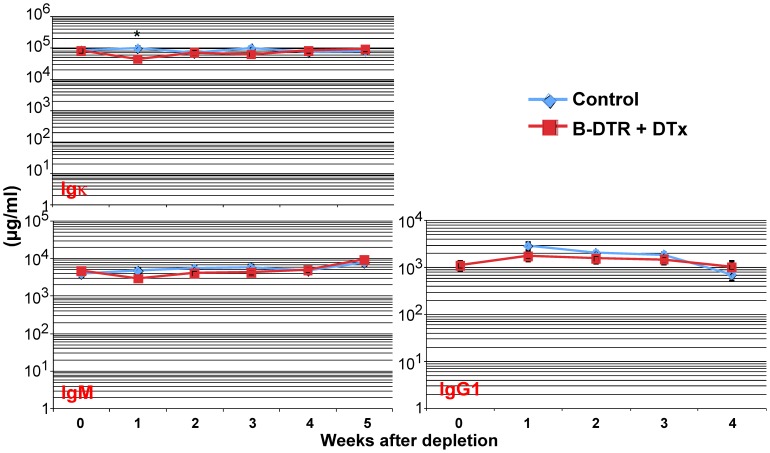
Total serum immunoglobulin levels after depletion of B cells. B-DTR mice and control mice were injected daily four times with 25 ng/g body weight DT. Mice were bled just before DT injection and in weekly intervals thereafter. Shown are the total immunoglobulin levels at the indicated time points. The values are shown as average of groups of at least five mice.

After showing efficient depletion of B cells using the B-DTR mice, we asked if B cell ablation would also influence the levels of immunoglobulins (Ig) in these mice. We therefore bled B-DTR and control mice at weekly intervals following DT application. As seen in Fig. 4, one week after depletion of B cells, we observed a transient reduction in total IgK isotype antibodies. Similarly, we found a reduction in the IgM and IgG1 levels in the sera of the DT-treated B-DTR mice compared to treated control mice (Fig. 4).

**Figure 5:**
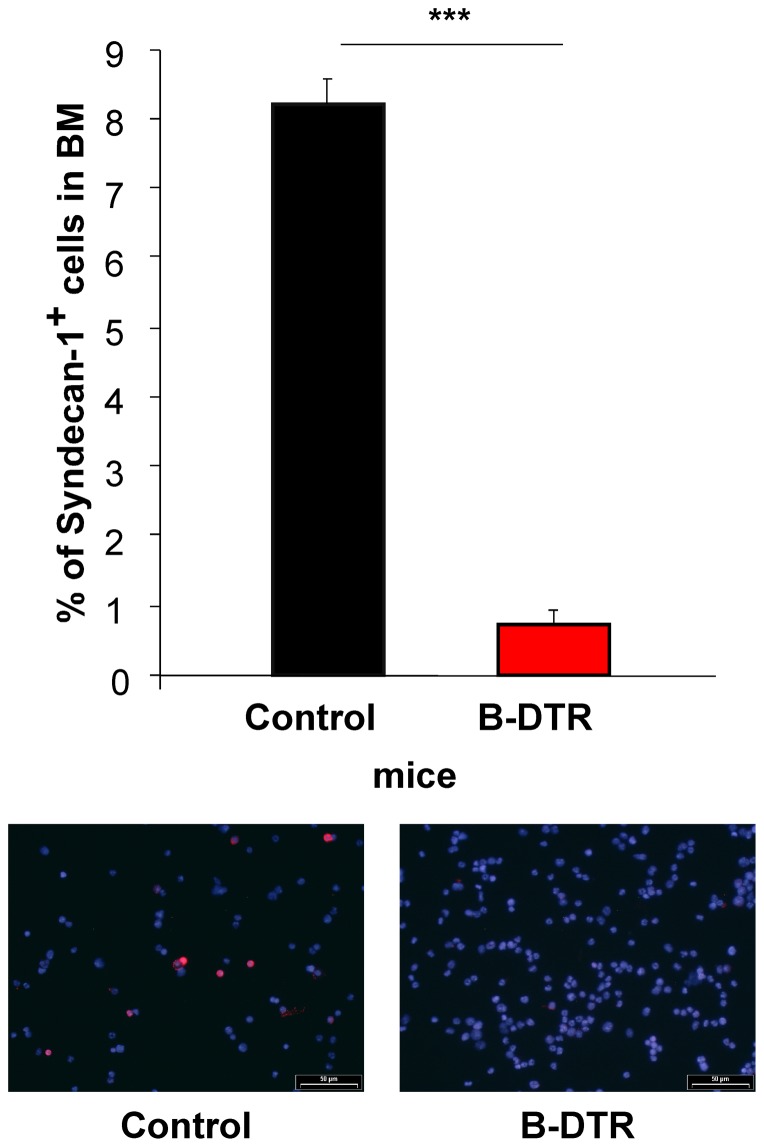
Plasma cells identified with Syndecan-1 in cytospins. Bone marrow cells from NP-CG immunized and boosted cells were analyzed for plasma cells. Plasma cells are shown in red with a surface staining of syndecan-1, the remaining cell population was counter stained with Hoechst (blue). The bar chart represents plasma cells found in the regarding groups (n = 5). The pictures show representative sections of the cytospins.

To test if plasma cells could be depleted also by DT application of the B-DTR mice, we immunized B-DTR and control mice with 4-hydroxy-3-nitrophenylacetyl coupled to chicken-gamma-globulin (NP-CG). Three weeks after NP-CG immunization, B-DTR and control mice were treated with DT. We analyzed the number of plasma cells in bone marrow seven days following antigen boost (four weeks after the depletion of B cells via DT treatment). Bone marrow cells were used to prepare cytospins that were stained for Syndecan-1 (CD138^+^ cells stained red). By counting syndecan-1^+^ cells, as compared to other BM cells, we found a significantly reduction in plasma cells from 8% in the BM of the control mice to just 1% left in the BM of the B-DTR mice (Fig. 5).

## Discussion

Although the first B cell depleting antibody was presented in 1984, no ideal system to deplete B cells and plasma cells was published so far. One limiting factor of existing depleting antibodies is availability and high costs. The B-DTR mice can serve as a relatively cheap and easy system to investigate B cell function in many diseases including rheumatoid arthritis, multiple sclerosis, systemic lupus erythematous, type 1 diabetes mellitus as well as in transplant experiments.

In our system although, we found good depletion in peripheral lymphoid tissues where hardly any B cells were left. In the bone marrow the depletion of B cells was less effective, comparable to the B cell antibodies used in clinical therapies. The efficiency of the depletion of B cells is also reflected in the expression of EYFP in the CD19-Cre reporter strain and the low effect found in bone marrow is a consequence of the early developmental stage of some B cells in this organ. This is emphasized, when the efficiency of the depletion of different B cell subpopulations in bone marrow was analyzed. Early B cells not yet IgM expressing are hardly depleted, while the B220^high^ IgM^+^ B cells are almost all depleted.

One unique and direct function of mature B cells is antibody secretion, but the mechanisms of regulation of the serum levels of antibodies are still unsolved. Therefore, it is not clear how B cell depletion affects the immunoglobulin levels. In the survey by DiLillo, serum immunoglobulins, Ag-specific Ab titers and the bone marrow Ab-secreting plasma cells numbers were not affected by CD20 antibody-mediated B cell depletion [29). In our system we found a marginal effect of B cell depletion on natural antibodies, but the effect on antigen-specific antibodies was more pronounced. Consequently our system seems to be more effective in depletion of B cells. Furthermore, antigen-specific antibodies are secreted by B-1 cells and plasma cells, consequently we have a strong evidence for plasma cell depletion as well. This can be explained by the advantage of the genetic modulation of the Cre-recombinase expressed in CD19^+^ cells, resulting in a genetic memory in the B-DTR mice. The consequence of this ‘memory’ is that also plasma cells, which loose their surface antigens due to a high membrane turn-over and stop CD19 expression, should still express the DTR and plasma cells despite their rare surface antigens and high membrane turn-over are DT sensitive. And consequently Plasma cell depletion in the bone marrow is occurring and was shown by cytospins. The low influence of the decreased number of plasma cells on the immunoglobulin level needs to be explained by the regulation mechanisms of serum immunoglobulin. The decrease in the plasma cell number of the bone marrow cytospins can be explained in two ways, first directly by depletion, secondly effective depletion of plasma cell progenitors.

An additional advantage of our inducible B cell depletion system next to availability and low costs is the possibility to investigate the optimal time point of B cell depletion for each disease, which profits from B cell depletion. Secondary effects of livelong B cell absence, like lacking IL-4 production by T cells [30), are excluded as well. It is possible to decrease the antibody specific serum level by B cell depletion although the depletion efficiency in bone marrow is low.

## Materials and Methods

### Mice

CD19-Cre/iDTR, CD19-Cre, iDTR all on C57BL/6 background, and wild-type C57BL/6 mice (Charles River) were kept in a specific pathogen-free barrier at the central animal facility of the University of Mainz and Regensburg. All experiments were done in accordance with guidelines of the Institutional Animal Care and Use Committees of the University of Mainz.

### Flow cytometry

Single cell suspensions were prepared from spleen, lymph nodes and bone marrow via passage through nylon cell strainers (40 µm). Red blood cell lysis of the single cell suspensions of spleen and bone marrow was performed with tris-ammonium chloride pH 7,2. Cells (1–10×106 per sample) were surface-stained in PBA with combinations of fluoresceine isothiocyanate (FITC), phycoerythrine (PE), CychromeTM (Cyc), allophycocyanin (APC) and biotin-conjugated monoclonal antibodies (mAbs) for 20 min at 4°C. Biotinylated antibodies were viszualized with Streptavidin conjugated to Cychrome. Antibodies specific to the following surface markers were purchased from BD Biosciences: B220; CD19; AA4.1; CD23; CD90.2; CD43; CD21; CD5; IgD; IgM and IgG1. All samples were acquired on a FACScan (BD Biosciences) and analyzed with the cellquest software (BD Biosciences). Absolute numbers of splenocyte subpopulations were calculated based on their percentage and the total number of splenocytes.

### Immunization and Depletion

8–10 weeks old control and CD19-Cre/iDTR mice were immunized by intra peritoneal injections of 50 µg NP-CG (4- hydroxy-3-nitrophenylacetyl chicken-gamma-globulin) in alum in a volume of 200 µl (Weiss and Rajewsky, 1990).

To deplete B cells a dose of 25 ng/g bodyweight was used in every DT injection. To get the best result DT was injected once a day intra peritoneal, 4 days in a row. In the course of the NP-CG immunization, the depletion of B cells was started 3 weeks after immunization.

### Bone marrow cells cytospins

To prepare cytospins, 2×10^5^ of Paraformaldehyde fixed B cells were centrifuged on glass cover slips resulting in cell spots and stored at −80°C.

The cells on glass cover slips were stained according to standard methods, using CD138 antibody (BD biosciences). All incubation steps during the staining procedure were carried out at RT. The cytospins were counterstained with Hoechst 33258.

### ELISA

Total serum immunoglobulins were analyzed via ELISA. In 96 well plates coated with the respective coating antibody for the requested Immunoglobulin. These plates were then incubated with the serial serum dilutions of the demanded time point after depletion.

### Statistics

Values are typically represented as mean ± SEM (standard error of mean). Statistical significance was assessed using 2-tailed Student's *t*-test. p-values <0.05 were regarded significant, displayed by ‘*’ in the figures (**  = p-value<0.05; ***  =  p-value<0.005).
